# Phenotyping bananas for drought resistance

**DOI:** 10.3389/fphys.2013.00009

**Published:** 2013-02-07

**Authors:** Iyyakkutty Ravi, Subbaraya Uma, Muthu Mayil Vaganan, Mohamed M. Mustaffa

**Affiliations:** ^1^Crop Production, National Research Centre for BananaTrichy, India; ^2^Crop Improvement, National Research Centre for BananaTrichy, India

**Keywords:** bananas, breeding, bunch yield, drought stress, phenotype, RWC

## Abstract

Drought has emerged as one of the major constraints in banana production. Its effects are pronounced substantially in the tropics and sub-tropics of the world due to climate change. Bananas are quite sensitive to drought; however, genotypes with “B” genome are more tolerant to abiotic stresses than those solely based on “A” genome. In particular, bananas with “ABB” genomes are more tolerant to drought and other abiotic stresses than other genotypes. A good phenotyping plan is a prerequisite for any improvement program for targeted traits. In the present article, known drought tolerant traits of other crop plants are validated in bananas with different genomic backgrounds and presented. Since, banana is recalcitrant to breeding, strategies for making hybrids between different genomic backgrounds are also discussed. Stomatal conductance, cell membrane stability (CMS), leaf emergence rate, rate of leaf senescence, RWC, and bunch yield under soil moisture deficit stress are some of the traits associated with drought tolerance. Among these stress bunch yield under drought should be given top priority for phenotyping. In the light of recently released *Musa* genome draft sequence, the molecular breeders may have interest in developing molecular markers for drought resistance.

## Introduction

### Importance of bananas

Bananas (refers to banana, plantain, and cooking bananas) are one of the earliest crop plants to have been domesticated. Originally, they were adapted from the humid tropics to broad subtropical climatic conditions. Bananas are one of the most important, but undervalued, food crops in the world. Bananas provide a staple food for millions of people; particularly plantains have remained a staple food of many ethnic groups in Africa, an area where the green revolution has had little influence. Bananas are considered an important food security crop, providing a cheap and easily produced source of energy. In addition, they are rich in certain minerals and in vitamins A, C, and B6. It has been estimated that the highest consumption rates are on the island of New Guinea and in the Great Lakes region of East Africa, where bananas form a large proportion of the diet and consumption amounts to 200–250 kg person^−1^ year^−1^ whereas in Europe and North America consumption is approximately 15–16 kg person^−1^ year^−1^ (INIBAP, 1992). Bananas are consumed in various forms, and consumption methods have evolved and been refined by humans over time. They are eaten raw, cooked, baked, steamed, or fermented. In many places, the whole plant is exploited with uses for the leaves, pseudostem, medicinally rich plant sap or fiber. Thus bananas and plantains are grown for specific purposes apart from the edible fruit and have become interwoven with the culture and livelihoods of human society. Although today bananas and plantains are best known as a food crop, almost every part of the plant can be used in one way or another.

### Cultivated area and yield performance under optimal conditions

Basically, bananas have occupied the status of commercial crop. Traditional banana growers, with the exception of a few large companies, are responsible for most production world-wide. Bananas and plantains (*Musa* spp.) are grown in more than a hundred tropical and subtropical countries and provide staple food for hundreds of millions of people. Bananas and plantains are grown in about 130 countries around the world, exhibiting a spectacular production of 122.85 million tons (FAOSTAT, 2012). India alone produces 26.20 million tons on an area of 0.70 million ha and contributes to 21.30 percent of global production (2007–2008). India is the largest producer in the world, followed by China, The Philippines, Brazil, and Ecuador. Around 87% of all the bananas grown worldwide are produced by small-scale farmers for home consumption or for sale in local and regional markets, while the remaining 13%, mainly dessert bananas, are traded internationally. Dessert bananas are also grown commercially in the subtropics and in Mediterranean climates including Israel and other East Asian countries, for internal consumption or local export (FAOSTAT, 2008). More than two-thirds of the bananas grown in the world for export are irrigated (Stover and Simmonds, 1987).

## Drought resistance

Water limitation is a major problem for global agriculture, permanently affecting 28% of the world's soils with almost half of all soils intermittently limited because of shallowness, poor water holding capacity, and other factors (Dudal, 1977). Drought in agriculture is “shortage of water in the root zone, resulting in decreased crop yield” (Salekdeh et al., 2009). Drought tolerance consists of drought avoidance and/or dehydration tolerance that are ultimately measured by the reproductive success of the species (Taylor et al., 2007). Drought avoidance strategies in plants include deep rooting, conservative use of available water to ensure grain filling is completed, and lifecycle modifications to match rainfall. Dehydration tolerance involves the plants' ability to partially dehydrate but remain viable and grow again when rainfall resumes.

The effect of drought on plants is complex and plants respond with many protective adaptations. Drought causes the plant to suffer from dehydration and overheating of its cells and tissues. Hence, drought resistance of the plant includes the ability to withstand dehydration and ability to withstand overheating (heat-resistant). High heat-resistance is not always linked with high drought-resistant and there is no universal mechanism of adaptation of plants to drought. Drought-resistance is a property which is formed and developed in the process of ontogenesis and is based on the whole preceding phylogeny of the plant. Based on the above observation, Henckel (1964) defined drought resistance as follows. “Drought-resistant plants are those which in the process of ontogenesis are able to adapt to the effect of drought and which can normally grow, develop, and reproduce under drought conditions because of a number of properties acquired in the process of evolution under the influence of environment.”

Drought is one of the important abiotic constraints restricting banana cultivation and its further adoption into non-conventional growing areas. Breeding for drought alone has not been focussed among any of the global banana breeding programs but it has been an essential trait considered along with other important ones like Fusarium wilt (race 1, 2, and 4), Sigatoka leaf spot (*M. fijiensis*, *M. eumusae*, and *M. musicola*), etc. Recent issues of climate change have warranted the need for the development of commercial banana varieties suited for less water environments. In this perspective the strengths and weaknesses in the banana crop for breeding drought tolerant genotypes has been discussed below with emphasis on genetic resources, drought tolerance, compatibility, outcome of breeding programs, etc. In this article explanation is placed largely on the basis of experiments performed in India.

### Bananas plant water relations

Bananas pose challenge to physiologists to measure indicators of water deficits, due to the presence of large air pockets within the leaves, and laticifers containing latex within the leaves, fruit, and corm that hinder the use of standard methods of measuring water relations (Turner and Thomas, 1998). Milburn et al. (1990), Kallarackal et al. (1990), Turner and Thomas (1998), subsequently demonstrated different methods to measure a series of physiological indications in relation to drought tolerance, *viz.*, of water potential, the volumetric (relative leaf water content), or thermodynamic tissue water status (leaf water, osmotic, and pressure potentials) of a laticiferous plant like the banana. The method described by Milburn et al. (1990), which is based on measurements of the refractive index of exuded latex, was preferred and its reliability subsequently confirmed by Thomas and Turner (2001). The water potential of well-watered plants was found to cycle diurnally within the remarkably narrow range of 0 to −0.35 MPa. In fact, the rate of extension of the youngest leaf may be the most sensitive indicator of plant water status (Kallarackal et al., 1990), providing it is not too hot (Thomas and Turner, 1998). Under hot, arid conditions, leaf folding is not considered to be a reliable plant-based indicator of when to irrigate (Thomas and Turner, 1998).

Banana production constraints are dominated largely by biotic and abiotic stresses. However, while research on biotic stresses has drawn sufficient attention worldwide, abiotic stresses have gone unnoticed. Among the abiotic stresses, drought, salinity and heat are the most important. Drought has rarely been addressed in the past, but is gaining importance in the face of depleting natural resources. The results of successful cultivation, especially of the water loving Cavendish clones, in drought prone areas with protected irrigation have provided the required momentum to perform research on drought in bananas. In subtropical and semi-arid banana cultivation zones, where rainy days are limited and there is an uneven distribution of rainfall, new crop management practices in terms of varieties selected, soil improvement (in terms of physical properties and nutrient enrichment), water management, etc. are being adopted. Although a large amount of research has been carried out on tropics including water management, drip irrigation, and fertigation, work on evaluation of banana and plantain varieties under conditions of water deficit is still very limited, as is the availability of related information. Probable reasons could be that most genebanks and breeding programs actively involved in germplasm evaluation and development are located in the humid tropics and ample rainfall. Moreover, creating large-scale drought conditions for a crop like bananas that is large and of long duration (12–20 months), presents many practical difficulties.

Screening germplasm for the drought has been initiated in some breeding programs such as that of the International Institute for Tropical Agriculture (IITA), Nigeria, NRCB, India and the Centro de Investigación Científica del Yucatán (CICY), Mexico. IITA has planted a large amount of germplasm in semi-arid zones of Uganda. The material that is being screened for drought tolerance includes landraces, East-African highland bananas, plantains and their triploid and tetraploid hybrids^1^. Similarly, NRCB is located in the dry tropics and is maintaining and evaluating a total of 340 core accessions for response to various biotic stresses, male, and female fertility, compatibility with other groups and subgroups, and seed setting ability. NRCB has screened 112 genotypes from a core collection of 340 accessions in response to water deficit conditions. Systematic screening of a wide range of germplasm for specific traits like leaf water retention capacity (LWRC) has also been attempted (Ravi and Uma, 2009). Observations on the response of various genotypes to water deficit under field conditions and their amenability for improvement through classical breeding are presented in Table 1.

**Table 1:** **General observations on germplasm performance under water deficit conditions and note on their breeding behavior (Anon, 1999, 2000, 2004, 2006, 2007; Uma and Sathiamoorthy, 2002; Uma et al., 2002)**.

**Genomic group**	**Subgroup or status**	**Genotypes (varieties/types)**	**Reaction to water deficit**	**Breeding behavior**	**General remarks**
AA	Wild	*M. acuminata* ssp Burmannica	Highly susceptible	Male and female fertile	Widely used donors for biotic stress tolerance genes
		*M. acuminata* ssp burmannicoides			
		*M. acuminata* ssp malaccensis			
		*M. acuminata* ssp zebrina			
BB	Wild	Types Athiakol, Manohar, Bacharia Malbhog	Susceptible	Male and female fertile	Not used in breeding program due to BSV being integrated into the host genome
		Types Attikol, Elavazhai	Less tolerant	Male and female fertile	Not used in breeding program due to BSV being integrated into the host genome
		Types Bhimkol	Moderately tolerant	Male and female fertile	Not used in breeding program due to BSV being integrated into the host genome
		*M. balbisiana* type Andaman	Tolerant	Male and female fertile	Not used in breeding program due to BSV being integrated into the host genome
AAA	Unique	Thellachakkarakeli	Moderately tolerant	Female fertile	Elite cultivars due to quality fruits. Produces average bunch even under water deficit conditions
	Cavendish	Grand Naine, Robusta, Dwarf Cavendish, Williams	Highly susceptible	Female fertile	Complete failure of crop
		Red banana and Green red banana	Susceptible	Moderately female fertile	Complete failure of crop
	Ney Poovan	Ney Poovan, Nattu Poovan, Njali Poovan	Tolerant	Reduced female fertile	Produces bunch even under water deficit
AAB	Mysore	Mysore, Poovan, Champa	Moderately tolerant	Female fertile	Produces bunch even under water deficit
	Pome	Prata	Susceptible	Female fertile	Fruits fail to fill and central core becomes conspicuous
		Small fruited varieties such as Pacha, Ladies Finger, Mannan, Krishnavazhai, Malai Kali	Susceptible	Female fertile	Fails to develop but under normal conditions sets seeds unlike counterparts with bigger fruits
ABB	Pisang Awak	Karpuravalli, Ankur-II, Gauria, Chinia, Bankela, Udhayam	Tolerant	Female fertile	Reduction in number of hands but retains finger size; sets seeds even under water deficit
	Monthan	Kachkel, Yengu Bontha, Bankeli, Pidi Monthan, Lamby	Moderately tolerant	Male fertile Female sterile	Reduction in number of hands and size of the fruit
		Ash Monthan	Tolerant	Male fertile Female sterile	Imposition of drought at flowering even with 3–4 green leaves produces normal bunch
ABB	Bluggoe	Birbutia, Bersain, Beula, Kothia, Chakia, Gauria, Nepali Kallu Monthan, Sakkai	Moderately tolerant	Male and female fertile	Bunches develop even with water stress; a hardy group of plants
ABB	Unique	Bangrier, Kanchikela	Moderately tolerant	Male and female fertile	Yield stability over the years; less reduction in yield; sets seeds even under water deficit, but has poor germination

Bananas, being a commercial crop in the tropical and subtropical region of the world, are prone to their growth and productivity being adversely affected by water stress. In traditional banana growing areas, long-term drought is not common, even though it is as potential an abiotic stress as short dry seasons. Inherent crop-based problems like being a long duration crop (10–12 months) make drought a potential threat in bananas. In addition, the high leaf area index (LAI) and shallow root system makes the banana plant extremely susceptible to water shortage (Robinson, 1996). Consequently the plant requires supplementary irrigations during dry periods to prevent reductions in yield and fruit quality. Some of the work carried out with bananas has been reviewed below, indirectly throwing some light on the crop's reaction to water deficit and field performance.

### Evapotranspiration from banana plantations

Precise information on the amount of irrigation to be applied is usually lacking, although a few experiments have been reported based on available water in the soil at field capacity. Depending on the prevailing climatic conditions, estimates of the annual evapotranspiration of the banana plant range from 1200 to 2690 mm (Robinson and Alberts, 1986). The water requirements of drip-irrigated bananas grown under semiarid conditions on a Mollisol or on an Ultisol with transient dry periods were determined. Using Class A pan factors that ranged from 0.25 to 1.25, it was found that all yield components for the plant crop and two ratoon crops were significantly improved with an increase in water application (Goenaga and Irizarry, 1998). Young et al. (1985) reported similar results when banana plants were irrigated according to pan factor treatments that ranged from 0.2 to 1.8. The water requirement of bananas in the humid tropics has been reported to be about 1–1.4 times the class A Pan evaporation (Stover and Simmonds, 1987). In a large-scale plantation in Honduras, plants were irrigated when the soil moisture tension (as recorded by tensiometers) exceeded −0.02 MPa at 15 cm and 30 cm (Stover and Simmonds, 1987).

### Banana genotypic variation for drought resistance

Banana plants are very sensitive to soil water deficit, as shown in numerous field experiments (Robinson, 1996). Banana leaves remain highly hydrated, even under drought (Shamueli, 1953; Turner and Thomas, 1998) indicating that the closure of stomata caused by soil water deficits is likely to be linked to a signal from the roots rather than a water deficit in the leaves (Turner, 2003). In a split root experiment, Thomas (1995) observed that drying part of the root system had no effect on leaf water status but did close the stomata. Severing the roots on the dry side caused the stomata to reopen. These observations support the view that the roots produce a signal that is transported to the leaves. This mechanism conserves the plant's water, but reduces carbon assimilation and productivity. From this point of view, study of root volume and structure may be less important. However, it is well-established that drought tolerant plants possess deep root systems. Root length density (Ld, measured in cm cm^−3^) and specific root length (Lw, measured in m g^−1^) are quantitative features of the architecture of root systems. Ld quantifies the capacity of the root system to explore the soil volume, and a high Ld means that the roots absorb more of the nutrients in a volume of soil, especially those nutrients that diffuse to the root surface. Banana and plantain roots have a Ld of about 1 cm cm^−3^ (Irizarry et al., 1981), which is similar to the root systems of trees. In contrast, Turner (2005) reported that herbaceous species have a Ld in the surface layers of the soil, of 4–50 cm cm^−3^. Therefore it is worth studying Ld, an important trait linked to drought stress in the banana root system, as described by Blomme et al. (2005).

In an experiment with cv. Williams has grown under subtropical seasonal conditions, plants that were well-watered in spring and autumn exhibited a high transpiration rate, especially with a normal summer. Whereas in extreme conditions of winter or a very hot summer, an internal stress developed within the plant, which reduced the transpiration rate in both situations. The evaporative demand exceeded the water absorption potential as reflected in decreased transpiration and stomatal conductance (Robinson and Bower, 1988).

However, there are not many reports on the impact of water stress at different growth phases on yield and yield parameters. In a field experiment conducted at the NRCB farm, water stress was imposed on plants under drip irrigation by withholding water for 1 month at flowering. This decreased the bunch weight by 42.07, 25.0, and 18.83 percent in cvs Robusta, Karpuravalli, and Rasthali, respectively. When water stress of 1 month's duration was imposed 30 days after flowering, the bunch weight was reduced by 18.83, 27.66, and 11.25 percent, and when imposed 60 days after flowering by 25.0, 16.84, and 16.47 percent, respectively in the three cultivars. Among all the three cultivars tested, Robusta was the most sensitive. The maximum reduction in fruit length (11–14 percent) and circumference (5.75–16 percent) was observed at harvest when water stress was imposed at flowering (Anon, 2008).

Banana cv. Williams in which bunch emergence occurred during a period of soil water stress (Ψs = −0.5 MPa) showed maturity bronzing at harvest, had shorter fruits and reduced green life (−29 percentage), and exhibited longer duration of fruit filling (Daniells et al., 1987). It has also been observed in bananas that growth and yields decreased drastically when the intervals between watering increased and when the soil moisture fell below 66 percent of the total available soil moisture (Robinson and Bower, 1988). Because of the tissue morphology, bananas require a certain amount of available soil moisture for normal development and growth. This high water requirement is the result of a large leaf area used for transpiration. It has been shown that the transpiration of banana plants in full sunlight is approximately 40–50 mg H_2_O dm^−2^ min^−1^ (Shamueli, 1953; Morello, 1954; Tai, 1977). According to previous calculations, the daily water uptake by cv. Dominico-Harton, a plantain with a constant leaf area of 14 m^2^ was estimated to be 26L in sunny weather, 17L in semi-cloudy weather, and 10L in cloudy weather. In a commercial plantation with a density of 1500 plants per hectare and a LAI of 2.1, water requirements were observed to be approximately 1170 m^3^, 765 m^3^, and 450 m^3^ in sunny, semi-cloudy, and cloudy weather, respectively. However, in practice, 150 mm of precipitation per month was reported to be sufficient to cover the water requirements of cv. Dominico-Harton (Belalcázar et al., 1990). In general, shortening of the irrigation interval with the same amount of water through a pulse system reduces the water tension in the upper soil layer, diminishes the soil temperature, encourages shallower rooting and reduces leaching of nitrates (Lahav and Kalmar, 1981). This practice is more important for the humid tropics and semi-arid areas.

Bananas are no longer considered as a single crop commodity owing to their vast diversity in terms of ploidy (2×, 3×, and 4×) and genomic constitution (AA, AAA, BB, AB, AAB, ABB, ABBB, etc.). Present day bananas have derived from two major ancestors *M. acuminata* contributing the A genome and *M. balbisiana* contributing the B genome. In nature, *M. acuminata* and its subspecies are considered as slender and delicate plants nurtured under shade and conducive environmental conditions, while *M. balbisiana* has diversified and being domesticated under harsh weather conditions, and is often resistant to many abiotic stresses including drought and extremes of temperature. On the other hand, banana cultivars containing the B genome being more resistant to abiotic stress than those solely based on the A genome. For instance, in Egypt where banana genetic diversity is higher than the rest of the region, the traditional AAB and ABB varieties cultivated in rural areas proved to be more resistant or tolerant to drought than the Cavendish ones (De Langhe, 2002). Another is the “Sugar” ABB Pisang Awak variety grown in Oman where it is shown to be well adapted to dryness at the Agriculture research station of Salalah (De Langhe, 2002). There are very few ABB dessert varieties showing good palatability and high productivity in the natural germplasm. Therefore, the triploid breeding strategy offers good future prospects through the combination of edible AB cultivars with wild *balbisiana* to create new productive dessert ABB varieties, palatable and tolerant to drought and cold temperatures.

*Musa* genotypes have exhibited differences in stomatal sensitivity based on the age of the leaf, and modulated by environmental factors such as irradiance, vapor pressure deficit (VPD), and soil-plant-water relations. On the basis of leaf conductance measurements, Ekanayake et al. (1994) identified tolerant ABB cultivars (“Fougamou” and “Bluggoe”) and sensitive genotypes (“Bobby Tannap” AAB and one of its hybrids TMP × 582–4) for transient dry conditions. In a pot study Thomas et al. (1998) compared the effects of environmental variables on leaf gas exchange processes (including transpiration) of three cultivars differing in their genomic constitution (“Williams” AAA; “Lady Finger” AAB; “Bluggoe” ABB). They found that, as the saturation deficit of the air was increased (from 1.5 to 5.7 kPa), both stomatal conductance and net photosynthesis declined linearly. Since increasing proportions of the B genome reduced this sensitivity to the dryness of the air and increased the instantaneous water use efficiency of the leaf, Thomas et al. (1998) concluded that the B genome contributes to drought tolerance in *Musa* spp.

*Musa* genotypes have different inbuilt mechanisms for resistance to drought stress. Research has been carried out on the effect of water deficit on commercial cultivars by a number of workers (Cayón et al., 1998), diploid *acuminata* clones (Ismail et al., 2000; Shamsuddin et al., 2000), and Cavendish clones (Eckstein and Robinson, 1995; Ramcharan et al., 1995; Orjeda and Suarez Sanchez, 1998; Thomas et al., 1998). However, there are very few reports on reactions to drought across the genotypes and their differential physiological, biochemical, and agronomic expression (Garcia and Manzanilla, 1994; Bananuka et al., 1999; Wagner et al., 2000; Abeywickrama and Weerasinghe, 2002; Ravi and Uma, 2009). Cultivars that demonstrated small reductions in gas exchange and leaf area and maintained the high water retention capacity and assimilation rate showed more resistance to drought stress (Bananuka et al., 1999).

Water stress induces oxidative damage and protective mechanisms differ among banana cultivars (Chai et al., 2005). Correlations between stomatal conductance, transpiration, and photosynthesis in water-stressed plants are well documented (Kallarackal et al., 1990). Twenty-four diploids (AA) were phenotyped for drought tolerant traits. A wide variation in chlorophyll content was found among the 24 diploid (AA) banana genotypes. In this group, Anaikomban recorded the highest chlorophyll a, chlorophyll b, and total chlorophyll content among all the genotypes tested and Hatidat, Kanaibansi, Siguzani, and Namarai recorded the lowest chlorophyll content (Anon, 2008).

## Methodology

Existing banana improvement programs have used only a fraction of the genetic diversity concealed in the wild and edible *Musa* species (*M. balbisiana and M nagensium*) (Lusty, 2005). Studies have been preliminary and neither exhaustive nor conclusive in terms of methodology, parameters, and research conditions. Much scope is left for future work to further refine the procedures and methodologies to be followed in the field as well as under controlled conditions.

### Breeding strategy

Bananas require frequent irrigation to avoid significant crop losses, especially during dry periods. Bananas, originally a tropical fruit crop, have reached the sub-tropics and even semi-arid regions owing to their adaptations and to growers' perseverance to manage the crop at the small expense of yield and quality. Modified or improvized agricultural practices like drip or fertigation over natural irrigation and flooding, and adaptation of banana varieties with a capacity to tolerate water deficits to a certain extent have allowed successful banana cultivation in non-conventional zones. However, breeding bananas for drought tolerance is an important alternative strategy to combat production constraints such as dwindling water resources.

Drought tolerance in bananas is the ability to survive under water scarcity during various stages of crop growth, without significant yield reductions. However, in nature, drought tolerance is always at the cost of yield and quality. Water scarcity can be overcome by cultural management or through genetic improvement. The latter is a long-term solution that can reach the poor grower and allow expansion of the crop to marginal lands. The technology to be developed has to be a robust and reproducible, with easy application in the field. The protocol for developing such technologies needs background information on various aspects of the crop in question—bananas—and a standard procedure. Earlier works on drought tolerance in bananas and drought as a trait have, for various reasons, been the subject of little research.

Drought is a complex environmental factor that is varied over a location and time frame. This makes it difficult for the researcher to create a standard for drought when the crop is challenged under field conditions. These realtime unpredictable situations are entirely different from screening for drought under controlled conditions. Therefore, breeding for the targeted environment with specific to the phenological stage will pave the success in breeding.

Reports in many other crops have suggested that the response to drought is a complex trait controlled by a number of genes. Drought seldom occurs by itself. In natural situations, drought is always coupled with high temperature stress and often with soil salinity. Working on a single mechanism to tolerate drought alone does not offer a solution; it needs to be researched with a multiple trait perspective. It is more complex in a crop like bananas because: (1) it is a long duration crop ranging from 12 to 18 months according to cultivar; and (2) it has three to four critical periods of crop growth spread over the 12–18 months, namely the juvenile stage, flower bud differentiation, shooting, and finally bunch maturity.

### Inherent problems in banana breeding

Most important of all, bananas have their own crop-based inherent problems for breeding, being a sterile crop (male and/or female), exhibiting polyploidy, and with most of the commercial varieties being sterile triploids and vegetatively propagated.

**Figure 1:**
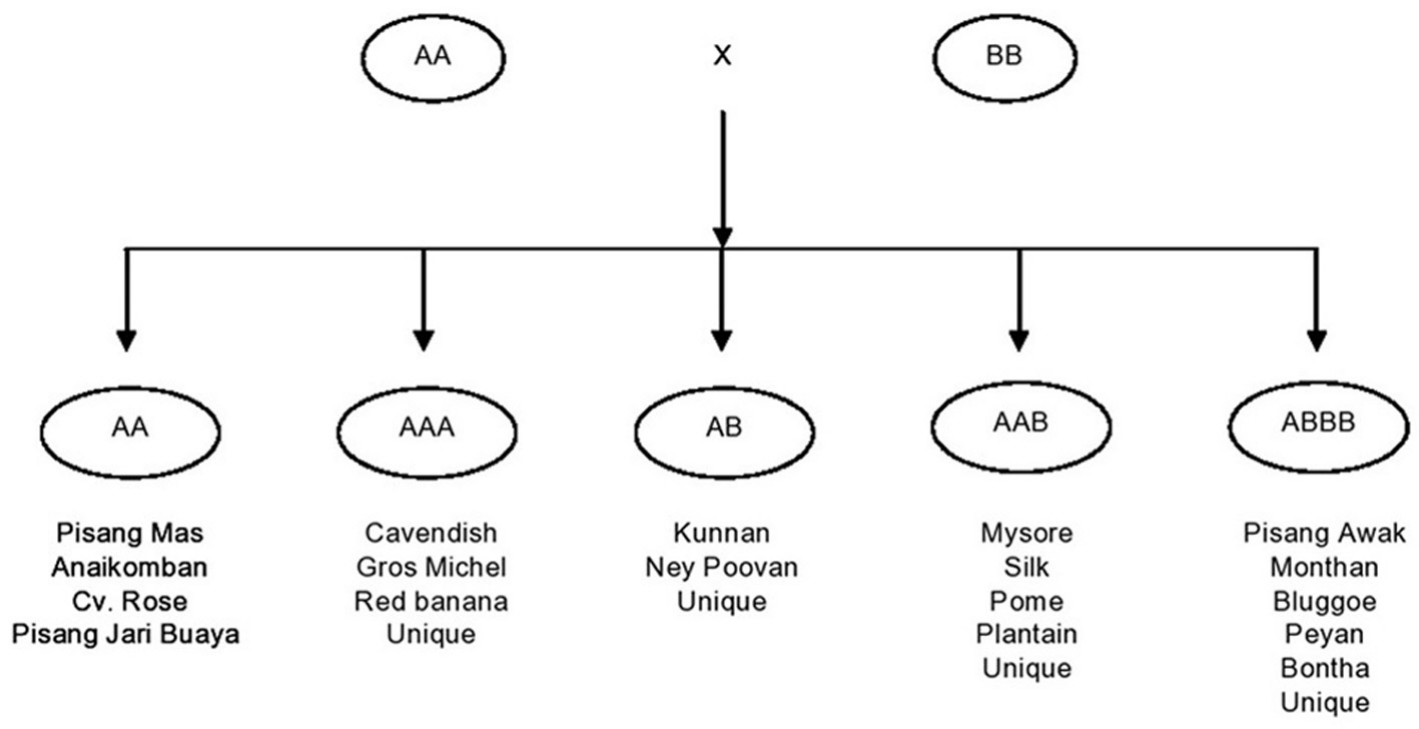
**Development of banana cultivars with the various major genomic groups**.

Another factor from the breeder's point of view is that bananas are no longer considered as a single crop commodity, since they have broad user applications. There are more than 30 varieties under cultivation in various parts of the globe. Each variety has its own breeding constraints. In the following section, breeding is addressed by the group and subgroup of economic significance. Present day commercial varieties of bananas and plantains are the evolutionary derivatives of crosses within and between two ancestral diploid species, the *M. acuminata* (AA) and *M. balbisiana* (BB) contributing A and B genomes, respectively (Simmonds, 1962). Although the involvement of other wild species such as *M. textilis* and *M. schizocarpa* has been proven (Carreel, 1994), a major role has been played only by *M. acuminata* and *M. balbisiana*. In conjunction with chromosome restitution, crosses have led to the development of autoploids and homogenomic hybrids, and alloploids and heterogenomic hybrids. Ploidy and genomic configurations played a vital role, leading to the development of the major groups being: diploids (AA, BB, and AB); triploids (AAA, AAB, and ABB); and tetraploids (AAAA, AAAB, AABB, and ABBB). Of these, commercial varieties fall into the genomic categories AAA, AB, AAB, and ABB. However, the cultivars grouped in the same genomic category could be very diverse (Simmonds, 1962; Stover and Simmonds, 1987) and hence, classified as subgroups. They consist of clones with similar morphological traits, having arisen from a single core clone through somatic mutations. Accordingly the classification of bananas is depicted in Figure 1.

Bananas and plantains are peculiar crops owing to their morphology. A sound breeding strategy needs background information on various fundamental aspects. In bananas, differentiation of the vegetative phase into the reproductive phase occurs with the completion of the emergence of all leaves. The number of leaves is a predetermined factor and ranges from 35 to 72, depending on the variety. The number of days taken to complete leaf emergence depends on the phyllochron values, which in turn depend on the prevailing climatic conditions. The normal range is from 6 to 16 months in *Eumusa* and can be as long as 50 months in some *Ensete* species at higher altitudes. Cessation of the vegetative phase is marked by the emergence of a flag leaf. On average, triploid commercial varieties take 8–12 months to complete the vegetative phase.

The flower axis start from the heart and pushes upward through the pseudostem. Generally, female or pistillate flowers are formed first on the flowering axis, followed by male or staminate flowers. Occasionally, the formation of perfect flowers in between the male and female phase is also noticed in some varieties. Flowers are borne on a cushion-like structure, arranged spirally and spatially along the axis. They are biseriate in nature and subtended or covered by a bract. Two or three bracts lift at a time and lifting starts from late evening to early morning. Female flowers become receptive in the early morning before the sun gets too hot. Anthesis have also started in the evening, and mature viable pollen is ready and available in the morning for crossing. In some genotypes, pollen germination was noticed before anther dehiscence. In such cases the time of crossing need to be carefully adjusted to achieve better results.

Pollen from the pollen parents is collected along with the flower. The pollen is squeezed out of the pollen sac using the thumbnail and is spread onto the receptive sticky stigma to effect pollination. The bunch is then covered with a muslin bag to prevent unwanted pollination. The ovary starts enlarging and develops into a fruit. In general, bananas are parthenocarpic, and fruits develop mainly from the ovary wall. This phenomenon does not need the stimulus of pollination.

Fruits are carefully collected after full maturity and allowed to ripen. Seeds are extracted either manually or mechanically. Seeds are freed from the adhering pulp and soaked in fresh water for 4–5 days, changing the water daily. On the fifth or sixth day, seeds are either sown in seed pans with sterile soil and coco pith mixture for better water retention. Seed germination is a highly variable factor depending on the variety and parental combination, but it ranges from 2 to 10%. The germination time varies from seven to 120 days. During this period, care should be taken to prevent seeds being eaten by ants and squirrels, or rotting due to soil-borne or water-borne fungi. When the seeds have germinated and two to three leaves emerged, the seedlings are shifted from pans to polybags with a red soil/sand/coco pith mixture. After 2 months, the plants become physiologically mature enough for field planting. Labeling is important at every stage.

#### Embryo culture and embryo rescue

Germination and successful regeneration of seed progeny is as low as <1 percent. Complementing seed germination with embryo culture can enhance the regeneration rate by 30–50 percent (Rowe and Rosales, 1996; Tenkouano, 2006). A protocol to improve germination through embryo rescue has not yet been well documented. However, Ortiz et al. (1995) have successfully extracted 55–60 percent mature embryos (instead of fully mature embryos at fruit maturity) and cultured them on half strength Murashige and Skoog (MS) medium with modified hormonal concentrations and under continuous light.

### Breeding schemes

#### Diploid breeding

Diploid breeding is vital to banana improvement programs, offering various advantages including a vast genetic background, the occurrence of high levels of male and female fertility, low levels of heterozygosity (which reduce the time to develop homozygous lines), easy genetic manipulation, and ease of study. Selected diploids, especially those exhibiting drought tolerance, are intercrossed to develop superior diploids, followed by selection for progenies exhibiting combined traits of drought tolerance and agronomic superiority.

#### Triploid–diploid breeding

The success of the triploid by diploid crosses depends on: (1) female fertility of the triploid; (2) the number of functional, fertile female gametes; and (3) inclusion of the B genome in either of the parents, and more specifically the triploid. The improved diploids are used to develop 4× and 2× progenies from 3× to 2× crosses. Alternatively 2× − 2× crosses may also result in 3× hybrids through unilateral sexual polyploidisation, where the parents produce either 2n pollen or a 2n egg (Tenkouano, 2006). Although some of the 4× progenies exhibit traits of interest, their female fertile nature results in the presence of seeds in the pulp, reducing consumer preference. This is overcome by crossing them with improved drought resistant diploid parents to derive sterile triploids (3×). Triploids are always superior to tetraploids in terms of sterility, reduced crop duration, optimum tree geometry, and better leaf retention. However, the choice of parents should allow capitalization on heterosis and pyramiding of genes of interest (Tenkouano, 2001). Although a number of breeding schemes can theoretically be conceptualized and attempted, the genomic and ploidy diversity in bananas makes the situation complex (Vuylsteke et al., 1997). The early success of the above mentioned breeding schemes makes them more realistic and practical, keeping in mind the complex nature of the drought tolerance trait.

#### Ploidy and genome analysis of progenies

Wide arrays of genotypes are observed in segregating population as a result of the variable ploidy and genomic status of the parents. Early analysis of ploidy and the genome is necessary to evaluate progenies for their basic purposes as dessert, cooking, or beer bananas. This is facilitated by the use of precise, non-destructive, faster and less labor-intensive flow-cytometry (Dolezel et al., 1994). For genome analysis, A- and B-specific markers have been developed and used (Pillay et al., 2000; Nwakanma et al., 2002). It has advantages over Simmonds' scoring system (Simmonds, 1966), by not relying only on morphotaxonomic traits and by being applicable at any stage of plant growth (Tenkouano, 2001). Genome-specific markers have been successfully employed in several breeding programs.

### Trial planning

#### Pot studies

Irrespective of whether it is the parent genotype or the hybrid progenies that are to be evaluated for drought, uniform planting material is a prerequisite. Being clonally propagated, *in vitro* culture offers bananas the best way to have uniform plants in sufficient numbers. Secondary hardened plants with 5–6 healthy leaves and a minimum of 4–5 major roots are selected for planting in 70–80 kg capacity concrete pots. The pots are filled with equal amounts of soil, sand, and compost. Fertilizer, NPK (15:15:15) is applied in doses of 20 g per plant before the induction of water stress The plants are irrigated regularly (on alternate days or by drip irrigation) until they are ready for imposition of the stress treatment. They are grown in a glasshouse or the phytotron where near-natural conditions are simulated. The stress period imposed through withholding of irrigation must be a minimum of 3–4 weeks to allow expression of the potential to adapt to the drought environment.

#### Field studies

For the field trial, uniform size suckers of recommended weight (1.5–2.0 kg) should be planted or tissue culture plants with 4–6 healthy leaves and 4–5 primary cord roots should be taken to achieve uniformity in growth and development (Ravi and Uma, 2011). Care should be taken to undertake this field trial in soil that is uniform in physical structure and fertility.

In the case of hybrid progenies, individual plantlets developed through embryo germination are planted in the field after 3 months under various hardening treatments. This is a pre-evaluation plot used for preliminary evaluations. Suckers obtained are multiplied *in vitro* for the production of 20–25 plants. From 10 uniform plants selected for screening against drought under controlled conditions. The actual number of plants required depends on the trait that is going to be studied and the methodology of the study. If the sampling technique is destructive, then more plants have to be made available when designing the experiment. During the crop growth period, side suckers should not be allowed to develop. One follower sucker should be allowed only after shooting. The plot should be weed free, and mechanical intercultural operations should be kept at a minimum to avoid root damage.

#### Water stress management and characterization

For evaluating any genotypes under field conditions, it is important to maintain cultural practices that are recommended in particular agro-climatic conditions as being most suitable for normal growth of the plant. The duration of stress is an important factor. Since the crop growth period extends over more than a year, the test accessions need to be protected from natural rainfall. For this purpose, a rainout shelter must be erected in the field and irrigated provided by a controlled irrigation system (drip/micro irrigation in the root zone). The size of the rainout shelters has to be determined according to the number of accessions and their maximum height. Estimating drought resistance in terms of the yield difference between potential (optimal) and stress conditions can differentiate genotype performance (Blum, www.plantstress.com).

As mentioned earlier, the stress intensity and phenological stage have to be defined based on the target environment. Though the bananas are sensitive to water stress, the most critical stage is the floral primordial initiation stage (Robinson and Alberts, 1986) than vegetative and fruit development stage. Water status measurements based on soil or root properties are more closely associated with leaf gas exchange than conventional techniques for measuring leaf water status (Turner and Thomas, 1998). The use of plant morphological characteristics in assessing plant water status, such as the rate of emergence of the youngest leaf also should not be ignored (Turner and Thomas, 1998). Thus, during the treatment period, soil matric potential monitored along while measuring leaf gas exchange parameters. The banana plant develops severe water deficit symptoms, when soil moisture reaches at *ca*. Fifty-five to sixty percentage of available soil moisture and then stressed plants must be given normal irrigation until the end of the harvest. To assess the effect of soil moisture deficit stress on juvenile vegetative stage, floral primordial initiation (PI) stage, flowering and bunch development on yield and yield parameters, separate experiment is to be laid out for each phenological stage. In banana cv. “Williams” early juvenile vegetative stage is insensitive to drought and 4–5 months after planting (coincides with PI stage) is sensitive to drought stress (Robinson and Alberts, 1986).

In places where a rainout shelter facility is not available, then the experiment has to be conducted in an arid zone where irrigation should be provided artificially. All the above parameters can be measured in field-grown plants, where the main concern is the overall effect of water stress on crop yield.

### Water and plant water strategy

In general, it is agreed that crop drought resistance is a major factor in the stabilization of crop performance in drought-prone environments. Drought resistance is now considered by breeders and molecular biologists to be a valid breeding trait. However, there is a serious lack of conceptualization, direction, and protocol for measuring drought resistance (Blum, www.plantstress.com). Tests for drought resistance must be performed with whole plants and/or plant communities (Blum, www.plantstress.com). Three major characteristics that contribute to genetic variation for drought resistance are: (1) maintenance of a high plant water content and delayed symptoms of water deficit such as wilting; (2) maintenance of plant function at a low water status; and (3) recovery of hydration and function from a low plant water status. The following methods accommodate the above points.

#### Phenotyping traits

Worldwide there is great interest in improving the drought tolerance of crop plants. Although it is known that drought adaptive traits are complex and multigenic, understanding of their physiological and genetic basis is incomplete, making specific genetic targets rare. Genetic improvement of drought resistance in crop plants require identification of relevant drought resistance mechanisms and the development of a suitable methodology for their measurement in the screening of germplasm or breeding population (Blum and Ebercon, 1981).

***Plant growth.*** Plant growth is the real measurement because the whole plant is involved in the comparison among treatments. Besides plant height, pseudostem girth, phyllochron and leaf emergence rate, the following growth parameters should be measured.

Plant growth analyses are performed as follows. Five plants are to be harvested at fortnightly/monthly and divided into their respective parts and dried at 70°C in hot air oven for 48 h, giving dry weight (W). The area of each leaf was calculated from the formula (Turner, 1972).

**Figure 2:**
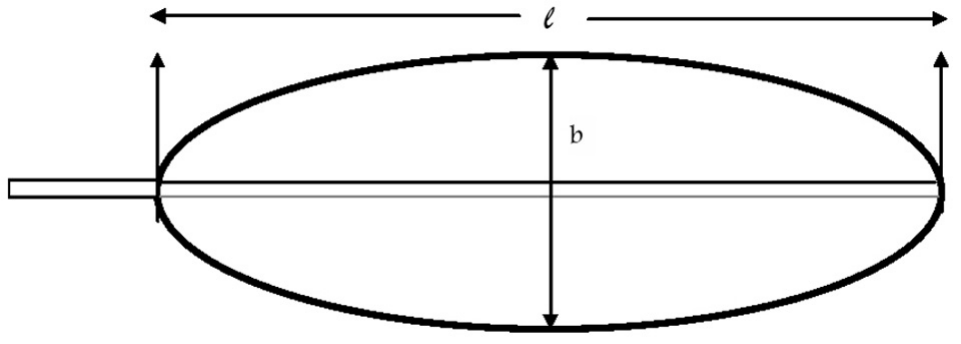
**Diagram representing the banana leaf for measuring lamina length (*l*) and width (*b*)**.

1. Leaf area (*A*) = 0.83 (*l* b) where *l* = length of lamina in cms and *b* = breadth of lamina at its widest point (Figure 2) (Summerville, 1944).

From these data four factors were calculated as follows:

2. Leaf Area Index *(L)*: area of leaf (*A*) per unit area of land (dimensionless).
3. Leaf Area Ratio (*F*): area of leaf (*A*) per unit of total plant dry weight (m^−2^ g^−1^).
4. Relative Growth Rate (*R*): (lnW2 – lnW1)/(t2 – t1), where *W*, and *W*, are plant dry weights at times *t*, and *t*, respectively.
5. Net Assimilation Rate (*E*): from (1/A). (DW/dt), where *W* is the dry weight of the plant at time *t* and *A* is the leaf area (g m^−2^day^−1^).
6. Specific leaf weight (SLW) is calculated as leaf area/leaf weight.
7. The leaf emergence rate (LER) is a useful index of the vegetative development rate of a banana plant and is closely related to temperature. The leaves emerged during the experimental period are noted in both control and treated plants. The total number of fully opened leaves produced during the experimental period should be calculated on a weekly basis.

Allometric relationships developed for highland banana (Nyombi et al., 2009) can be adopted in all other environments by evaluating different phenological stages for use in growth assessment, understanding banana crop physiology, and yield prediction. An allometric relation is one whereby one measured parameter is a good estimate of other unmeasured parameter in the same organism. The authors derived the following equation to derive various growth parameters for highland bananas. Total plant leaf area (TLA) was estimated as the product of the measured middle leaf area (MLA) and the number of functional leaves. MLA was estimated as MLA (m^2^) = −0. 404 + 0.381 height (m) + 0.411 girth (m). The allometric relationship between aboveground biomass (AGB in kg DM) and girth (cm) during the vegetative phase followed a power function, AGB = 0.0001 (girth) 2.35 (*R*^2^ = 0.99), but followed exponential functions at flowering, AGB = 0.325 e0. 036 (girth) (*R*^2^ = 0.79) and at harvest, AGB = 0.069 e0.068 (girth) (*R*^2^ = 0.96). Girth at flowering was a good parameter for predicting yields with *R*^2^ = 0.7 (cv. Mbwazirume) and *R*^2^ = 0.57 (cv. Kisansa) obtained between actual and predicted bunch weights. This article shows that the allometric relationship can be derived for different banana cultivars in different agroclimatic zones for developing banana growth models, which can help breeders and agronomists to further exploit the crop's potential.

***Plant water status***

*Relative water content*. The relative water content (RWC) is the ratio of water present in the leaf disc at the time of sampling to that present in the excised disc after it has been fully dehydrated. The result is expressed as a percentage. A concern about this technique is the absorption of water into the intracellular spaces in the floating discs (Milburn et al., 1990). The values of RWC obtained in the study by Turner and Lahav (1983) revealed absorption of water into the intracellular spaces not to be a dominant factor. Therefore, this parameter needs to be considered to assess drought resistance traits. The third youngest leaf is taken for this measurement. Twenty leaf discs (12 mm dia) are extracted with a cork borer, half of the number from each half of the middle place of the lamina and weighed immediately, taking care to minimize water loss from the fresh sample. Discs float on deionized water containing CaSO_4_ for 6 h at 25°C, and then weighed to determine the turgid weight. The leaf discs are placed between two layers of tissue paper with a 500 g weight placed on it for 1 min before weighing (Weatherly, 1950; Barrs, 1968). The leaf discs are then oven dried at 105°C for 24 h. RWC is calculated as follows:

$$\text{RWC}=\text{1}00\left(D_{f}-D_{d}\right)/\left(D_{t}-D_{d}\right)$$

where, *D*_*f*_, *D*_*t*_, and *D*_*d*_ are the fresh, turgid, and dry weights, respectively. The RWC data recorded in banana genotypes with different genomic background is presented in Table 2. Where, Drought stress was imposed at 6-months-old plants for 3 weeks and the soil matric potential reached at −0.60 MPa at the end of the stress period.

**Table 2:** **Relative water content (RWC) of drought stressed banana genotypes**.

**Genotypes with genome background with ploidy level**	**0 DAT**		**7 DAT**		**14 DAT**		**21 DAT**		**28 DAT**	
	**T1**	**T2**	**T1**	**T2**	**T1**	**T2**	**T1**	**T2**	**T1**	**T2**
Paghalapahad wild (BB)	93.2	90.8	94.4	79.3	81.0	67.3	76.3	69.3	78.7	71.7
Athiakol (BB)	74.3	68.2	86.5	80.4	78.9	70.7	85.2	63.8	87.6	66.2
Karpuravalli (ABB)	81.0	83.3	79.7	71.2	79.3	70.4	87.2	61.9	79.5	64.3
Peyan (ABB)	81.2	77.0	81.2	69.8	87.6	68.4	76.2	62.6	78.6	65.0
Kothia (ABB)	97.5	92.0	87.5	69.0	83.5	68.5	85.6	62.8	88.0	65.2
Vennutu Mannan (ABB)	82.8	81	82.7	70.23	85.0	78.2	90.9	83.6	93.2	86.0
Saba (ABB)	81.2	78.5	89.7	79.8	85.7	75.7	77.3	84.5	79.6	66.9
Monthan (ABB)	76.3	78.9	81.1	78.2	78.9	76.3	85.6	86.7	87.9	69.1
Nendran (AAB)	77.0	79.8	78.2	73.9	81.4	71.5	84.3	71.2	86.6	73.6
Poovan (AAB)	80.1	88.8	93.3	71	81.7	76.9	84.7	75.9	87.0	68.3
Chinali (AAB)	83.2	79.6	88.6	72.3	75.9	68.6	86.9	71.8	89.2	74.1
Rasthali (AAB)	86.5	80.8	81.7	72.6	79.8	60.8	86.9	68.2	89.2	70.6
Jwari Bale (AAA)	81.4	81.2	81.23	62.4	89.0	70.0	87.7	56.2	90.1	68.6
Ney Poovan (AB)	82.4	85.8	88.4	69.2	79.0	69.0	87.4	63.8	79.8	66.2
Robusta (AAA)	90.2	88.0	83.56	67.9	80.4	71.4	83.0	56.4	85.4	68.8
Red Banana (AAA)	87.7	78.8	81.32	76.1	76.9	77.9	88.7	52.6	91.1	65.0
Pisang Jari Buaya (AA)	60.6	53.5	85.14	70.1	83.4	64	85.6	50	87.9	62.4
Calcutta 4 (AA)	75.9	75.9	81.21	71.1	79.3	64.4	84.9	69.6	77.2	72
X	81.81	80.11	84.75	72.47	81.48	70.56	84.69	67.27	85.37	69.11
*T*	NS		^*^		^*^		^*^		^*^	
*V*	^*^		^*^		^*^		^*^		^*^	
*T* × *V*	NS		^*^		^*^		^*^		^*^	
CV%	10.68		10.6		13.31		9.64		10.72	

*Drought stress was imposed in 6-month-old plants*.

*Source: Ravi and Uma (2012)*.

* *Significance at 5% level of CD. NS, Non significant; T, Treatment; V, Genotype; T1, control; T2, stress; DAT, Days after treatment*.

*The relative water content (RWC) of banana genotypes significantly varied between treatments after the second week of stress imposition (Table 2). After 14 DAT the RWC in T2 maintained between 70.56 and 69.11. In irrigated control RWC maintained > 80*.

*Leaf water retention capacity*. Assessment of the rate of water loss from excised leaves or plants has shown some promise for differentiating drought resistance of wheat cultivars (Bayles et al., 1937; Sandhu and Laude, 1958; Salim et al., 1969; Dedio, 1975; Clarke and McCaig, 1982). A similar technique was applied by Bananuka et al. (1999) to assess drought stress resistance in bananas. The principle behind this technique is that drought stressed or hardened plants retain more water than unstressed plants and, when the stress is imposed across many different genotypes, the tolerant genotypes exhibit a greater capacity for water retention in the leaf tissue. The difference in LWRC may be due to the tightness of stomatal closure (Kirkham et al., 1980) or to other causes such as cuticular resistance to water loss (Clarke and McCaig, 1982). The third fully matured leaves from the top is sampled for measuring LWRC. Leaves (or strip of leaves) are excised and put under a polyethylene cover to avoid losing moisture and immediately weighed to give the fresh weight. They are left in a chamber at a temperature of 30–35°C and a RH of 50–60 percent for 24 and 48 h and weighed again. The leaves are also weighed after oven drying at 80°C for 24 h. Then the leaf water is calculated for 24 and 48 h by subtracting the oven dried leaves and expressed in terms of percentage of leaf water present at 24 and 48 h, as follows:

Leaf water retention capacity(%)=(fresh weight−dry weight)/  fresh weight×100

The LWRC, as a proxy for drought resistant or water-use efficiency, should be treated very cautiously (Turner et al., 2007). For large-scale field screening this method can be very well adopted (Ravi and Uma, 2009) and must be validated for yield under irrigated and stress plots so that drought resistance/susceptibility can be quantified.

***Plant function***

*Leaf gas exchange.* Leaf gas exchange or the rate of extrusion of the leaf is a more sensitive method for determining the response of banana plants to water deficit. A strong association exists between soil water status and leaf gas exchange (Turner and Thomas, 1998). This parameter can be measured with the portable photosynthesis measuring system, e.g., an infrared gas analyzer (IRGA). The third youngest leaf is used, with a minimum of three measurements for each leaf. The middle or distal quarter of the third youngest leaf is used for the measurement.

*Quantification of photosynthetic pigments.* Drought affects photosystem II more than photosystem I in the photosynthetic mechanism. They become uncoupled, resulting in free, high-energy electrons in the leaf. The uncoupled electron transport leads to photooxidation of chlorophyll and loss of photosynthetic capacity. The chlorophyll content is measured from the third youngest leaf lamina, sampling from both sides of the middle portion of the leaf from three plants, using three replications for each treatment. Banana leaf discs of 40–50 mg (3–4 leaf discs with a diameter of 10 mm) in fresh weight can be extracted in 10 mL of dimethyl sulphoxide (DMSO) in a glass test tube covered with aluminium foil and kept in an oven at 65°C for 4 h (Hiscox and Israelstam, 1979). Tubes are withdrawn and the temperature brought down around 25°C. Leaving the sample over night under dark in the room temperature (23–25°C) ensures complete extraction of pigments and in DMSO chlorophyll degradation is negligible. The optical density (OD) of the extract is read on a spectrophotometer at 645 nm and 663 nm and chlorophyll a (chl a) and chlorophyll b (chl b) concentrations (in μg ml^−1^) are calculated using the formula given by Arnon (1949):

$$\begin{array}{l} \text{Chl a}=\text{12}.\text{7D 663}-\text{2}.\text{69 D645}\\ \text{Chl b}=\text{22}.\text{9 D645}-\text{4}.\text{68 D663} \end{array}$$

*Cell membrane stability.* Tissue tolerance may be exhibited and measured in any of the tissue's physiological or metabolic functions. It is a process specific since different physiological processes may show tolerance or susceptibility (Blum, 1979). A valid and functional drought tolerance test should therefore relate to integrated plant responses at low plant organization level (i.e., tissue growth), or a single attribute related to the basic facets of cellular or tissue responses to stress (Blum, 1981). A critical role of cell membrane stability (CMS) under conditions of moisture stress as a major component of drought tolerance (Bewley, 1979). The rate of injury to cell membranes by drought is estimated through measurement of electrolyte leakage from the cells (electrical conductivity). For drought tolerance, the method is based on dehydration of leaf discs in PEG solution and subsequent measure of electrical conductivity of aqueous medium.

Banana genotypes are to be grown free from diseases and nutrient deficiencies. In two sets of plants per genotype, one set of plants is subject to soil moisture deficit stress by withholding irrigation and other will be irrigated. Twenty leaf discs (1.2 cm diameter) are to be taken from 3 to 4 plants per replication from third fully matured young leaf. The leaf discs are to be placed in 100 cm^3^ flask and washed 2–3 times with deionized distilled water. For desiccation treatment (T) leaf discs are to be submerged/float in 30 cm^3^ of 30% of PEG 6000 solution for 24 h at 10°C and for control (C) incubate the leaf discs with deionized distilled water. After incubation in PEG leaf discs are to be quickly washed three times with deionized distilled water. Both desiccated and control samples are to be immersed in 30 cm^3^ of deionized distilled water for 24 h at 10°C. The flask contents are then warmed to 25°C, shaken and electrical conductivity is measured using an Electrical Conductivity Meter. Following conductivity measurement, the leaf tissues are to be autoclaved for 15 min and again electrical conductivity is measured at 25°C. Sufficient replications are to be maintained to satisfy the statistical analysis.

CMS of leaf tissues is calculated as the percentage injury using the following equation.

Percentage injury = 1 − [1 − (*T*_1_/*T*_2_)/1 − (*C*_1_/*C*_2_] × 100, where *T* and *C* refer to mean of treatment and controls, respectively, and the subscripts 1 and 2 refer to initial and final conductivities, respectively.

*Yield and yield parameters.* Any stress effect ultimately has to be evaluated in terms of economic yield. Therefore, drought stress imposed on the third and fifth month after planting and at flowering for a period of 4 weeks has to be evaluated in terms of yield. In the Table 3, bunch yield of different banana genotypes under drought stress (imposed at 6-month-old plants for 3 weeks and soil matric potential reached −0.6 MPa at the end of stress period) is presented.

**Table 3:** **Effect of drought stress on banana bunch weight (*V* × *T*)**.

**Genotype treatment**	**LS mean**	**Group**	**Bunch weight (Kg)**
V7	T1	20.33	A
V5	T1	16.66	AB
V6	T1	15.19	ABC
V8	T1	14.16	BCD
V7	T2	13.87	BCDE
V2	T1	13.00	BCDEF
V8	T2	11.38	BCDEFG
V10	T1	11.00	BCDEFG
V1	T1	11.00	BCDEFG
V3	T1	10.67	CDEFG
V6	T2	10.58	CDEFG
V11	T1	9.67	CDEFGH
V12	T1	9.67	CDEFGH
V3	T2	9.33	CDEFGHI
V10	T2	9.27	DEFGHI
V9	T1	9.17	DEFGHI
V1	T2	9.00	BCDEFGHI
V15	T1	8.83	DEFGHI
V5	T2	8.67	DEFGHI
V14	T1	8.17	EFGHI
V11	T2	8.17	EFGHI
V16	T1	8.00	FGHI
V4	T1	7.83	FGHI
V9	T2	7.00	GHI
V17	T1	6.83	GHI
V2	T2	6.50	FGHI
V16	T2	6.33	GHI
V4	T2	6.33	GHI
V14	T2	6.17	GHI
V13	T1	6.00	GHI
V15	T2	6.00	GHI
V18	T1	5.90	GHI
V13	T2	4.50	HI
V18	T2	3.75	HI
V12	T2	3.50	I
V17	T2	3.50	HI
Paghalapahad wild (BB)			V1
Athiakol (BB)			V2
Karpuravalli (ABB)			V3
Peyan (ABB)			V4
Kothia (ABB)			V5
Vennuttu mannan (ABB)			V6
Saba (ABB)			V7
Monthan (ABB)			V8
Nendran (AAB)			V9
Poovan (AAB)			V10
Chinali (AAB)			V11
Rasthali (AAB)			V12
Jwari bale (AAA)			V13
Ney Poovan (AB)			V14
Robusta (AAA)			V15
Red banana (AAA)			V16
Pisang Jari buaya (AA)			V17
Calcutta 4 (AA)			V18

*Source: Ravi and Uma (2012)*.

**Figure 3:**
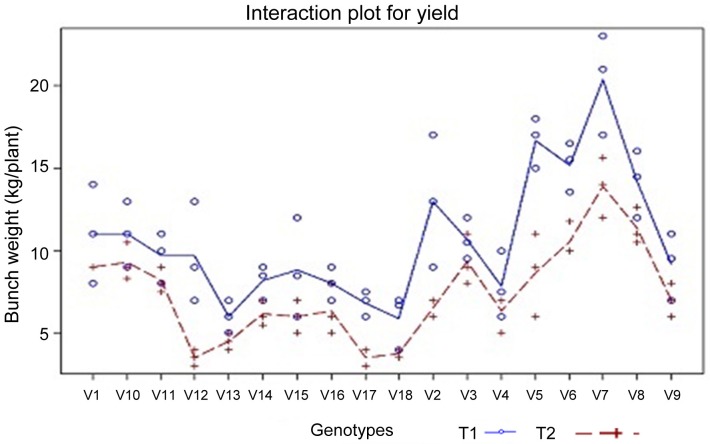
**Interaction plot for yield (bunch weigh in kg; *n* = 3) under different treatments (TRE), i.e., irrigated (T1-blue solid line) and drought stress (T2-red dotted line; soil moisture deficit stress imposed at six month old plants for three weeks and soil matric potential reached −0.6 MPa at the end of stress period) condition in different banana genotypes (VAR); V1—Paghalapahad Wild (BB); V10—Poovan (AAB); V11—Chinali (AAB); V12—Rasthali (AAB); V13—Jwari Bale (AAA); V14—Ney Poovan (AB); V15—Robusta (AAA); V16—Red Banana (AAA); V17—Pisang Jari Buaya (AA); V18—Calcutta 4 (AA); V2—Athikol (BB); V3—Karpuravalli (ABB); V4—Peyan (ABB); V5—Kothia (ABB); V6—Vennuttu Mannan (ABB); V7—Saba (ABB); V8—Monthan (ABB) and V9—Nendran (AAB).** Source: Ravi and Uma (2012).

The bunch weight of different genotypes under irrigated and stress environment analyses in SAS 9.2 Proc Glm model for Tukey test (Table 3). Among the tested banana genotypes Saba (ABB), Monthan (ABB), and Vennutu Mannan (ABB) recorded higher bunch yield under soil moisture deficit stress. These genotypes also recorded higher bunch yield under irrigated environment. The interaction plot for yield (Figure 3) also revealed the same.

Yield components such as the number of hands, the number of fingers, the rate of finger growth (in terms of length and circumference) must be compared in stressed and unstressed plants. At harvest, the individual finger weight difference can be calculated for the stressed and unstressed plants as:

$$\text{Geometric mean yield}\left(\text{GM}\right)=\left(Y_{s}\times Y_{w}\right)/\text{2}$$

where *Y*_*s*_ = genotypic performance under stress and *Y*_*w*_ = genotypic performance under well-watered condition.

*Drought susceptibility index.* The drought susceptibility index (DSI) is measured following Fischer and Maurer (1978):

$$S=\left[\text{1}-\left(Y_{s\;}/Y_{w}\right)\right]/DII$$

where, *DII* = (1 − *X*_*d*_/*X*_*p*_), and *X*_*d*_ and *X*_*p*_ are the mean experiment yield of all genotypes grown under drought stress and well watered regimes, respectively. In addition to yield and yield parameters, the traits measured in the preliminary evaluation also have to be taken into consideration for correlating with economic yield. For large numbers of genotypes (>25), the preliminary screening has to be done in the greenhouse or under a protected structure using plant growth analyses, plant-water relations, and plant functions. Drought tolerant plants identified thus must then be evaluated in the field. During field evaluation, stress can be imposed at different phenological stages.

## Conclusions

Water limitation is a universal problem for agriculture. Bananas and plantains are a staple food for developing countries and a high value crop in others. Increased production of bananas and plantains with limited resources, especially water, is a priority. This can be achieved through improved production technologies and varietal improvement for water-limited environments. Improvement of varieties through conventional and novel approaches relies on identification of traits conferring drought tolerance. Phenotyping bananas for drought resistance, in germplasm available with the national agricultural research systems across the world, is to be prioritized to identify useful accessions for the banana improvement program. Many published literatures support on soil water status is linked to leaf gas exchange parameters and rate of emergence of the youngest leaf. Measurement of the fraction of transpirable soil water (FTSW) is laborious in banana but valuable information can be generated as this variable can be used in much the same way that “leaf nitrogen (N) content” is used instead of “applied N fertilizer” as the independent variable in studies on the impact of N fertilizer on leaf photosynthetic rates (Peng et al., 1995). In general, any variation in experimental conditions during the imposition of water stress should be avoided. Care should be taken to control all other factors except the stress to be imposed, which is slightly difficult to manipulate. Cultivation and management are to be practiced as recommended for that test location. The stress has to be imposed for 3–4 weeks. Local drought tolerant varieties (based on their field performance) should be used as controls. The impact of the stress should be studied upto the final harvest. Any plant measurements must be corroborated with measurements of the soil moisture status. A controlled environment such as a rainout shelter is a preferred facility for conducting field level experiments in a long duration crop like bananas. It is an established fact that in bananas root signals plays a very significant role in recognizing the soil moisture deficit stress. The morphology, geometry, and quantitative nature of banana roots (root number, length, diameter, root mass, and RLD) merit study in relation to drought tolerance.

There is a great interest evinced among researchers in improving the drought tolerance of crop plants across the world. Presently, researchers are able to elucidate gene functions and mechanisms to regulate major plant traits through genomics, epigenomics, transcriptomics, proteomics, and metabolomics. To reap the benefit of recent molecular tools, good phenotyping is warranted. In the light of *Musa* genome sequence information available to all the researchers from July 2012, a quantum jump of molecular work toward the banana improvement program is contemplated.

### Conflict of interest statement

The authors declare that the research was conducted in the absence of any commercial or financial relationships that could be construed as a potential conflict of interest.

## Footnotes

^1^http://www.iita.org/cms/details/banana_project_details.aspx?articleid=228&zoneid=308
